# Social network characteristics are correlated with dietary patterns among middle aged and older South Asians living in the United States (U.S.)

**DOI:** 10.1186/s40795-020-00368-1

**Published:** 2020-09-11

**Authors:** Sameera A. Talegawkar, Nicola Lancki, Yichen Jin, Juned Siddique, Meghana Gadgil, Alka M. Kanaya, John A. Schneider, Linda Van Horn, Lawrence De Koning, Namratha R. Kandula

**Affiliations:** 1 Department of Exercise and Nutrition Sciences, Milken Institute School of Public Health, The George Washington University, Washington, DC, USA; 2 Department of Preventive Medicine, Northwestern University Feinberg School of Medicine, 420 E Superior, Rubloff Building 6th Floor, Chicago, IL 60611 USA; 3 Division of General Internal Medicine, Department of Medicine, University of California, San Francisco, CA USA; 4 Department of Medicine, University of Chicago, Chicago, IL USA; 5 Department of Pathology and Laboratory Medicine, The University of Calgary, Calgary, Alberta Canada

**Keywords:** Social networking, Diet, Dietary behavior, South Asians, Acculturation

## Abstract

**Background:**

Social and cultural norms, operating through social networks, may influence an individual’s dietary choices. We examined correlations between social network characteristics and dietary patterns among South Asians in the United States (U.S.)

**Methods:**

Data from the Mediators of Atherosclerosis in South Asians Living in America (MASALA) Social Network study were analyzed among 756 participants (mean age = 59 y standard deviation [SD] = 9 y; 44% women). A culturally adapted, validated food frequency questionnaire was used for dietary assessment. A posteriori dietary patterns using principal component analysis were named 1) animal protein, 2) fried snacks, sweets and high-fat dairy, and 3) fruits, vegetables, nuts and legumes. Social network characteristics were assessed using a standard egocentric approach, where participants (egos) self-reported data on perceived dietary habits of their network members. Partial correlations between social network characteristics and egos’ dietary patterns were examined.

**Results:**

The mean social network size of egos was 4.2 (SD = 1.1), with high proportion of network members being family (72%), South Asian ethnicity (89%), and half having daily contact. Animal protein pattern scores were negatively correlated with fruits and cooked vegetables consumption of network. Fried snacks, sweets and high-fat dairy pattern scores were positively correlated with sugar-sweetened beverages, South Asian sweets, fried/fast foods and *ghee* (clarified butter) consumption of network. Fruits, vegetables, nuts and legumes pattern scores were positively correlated with vegetables, fruits, and brown rice/quinoa consumption of network.

**Conclusions:**

Network member characteristics and their perceived dietary behaviors were correlated with dietary patterns of egos. Dietary intervention studies among South Asians should consider social network characteristics as candidate components for dietary intervention.

## Background

Dietary patterns and behaviors are important lifestyle factors for maintaining health and preventing chronic diseases [1]. Dietary patterns reflect the overall quality of diet and represent the complexity of dietary exposures currently influencing nutrition research and policy [2]. Recent federal dietary recommendations have emphasized improving overall dietary patterns beyond specific nutrients because dietary patterns account for interactions across food groups, foods, and nutrient density. Consuming a healthy dietary pattern has been associated with lower risk of many preventable chronic diseases [3–6]. Using a posteriori dietary patterns, a prudent dietary pattern was found to be significantly associated with lower risk of all-cause and cardiovascular disease mortality [7] and type 2 diabetes [8], and a pattern characterized by high intakes of fiber and low fat dairy was associated with less annual increases in Body Mass Index (BMI) and waist circumference [9]. These studies underlie the importance of maintaining overall diet quality by consuming dietary patterns associated with the maintenance of overall health.

Changing dietary behavior can be difficult because it requires attention to several factors at multiple levels including food availability and prices, existing knowledge of and attitudes towards healthy foods as well as social and cultural norms. It is therefore important to learn more about factors that are related to changes in dietary patterns and behaviors to make more effective recommendations and inform interventions [6, 10].

The Social-Ecological Model provides a framework for health promotion related interventions and includes an interplay between individual, relationship, community and societal factors, demonstrating a substantial role for social influence of networks on an individuals’ social and cultural norms and values which further affects food choices [6]. In recent years, the role of social networks in behavioral change has gained significant attention, wherein individuals may be influenced by their networks through conformity to social norms related to healthy behaviors [11]. Network members may be role models (or provide social control) for healthy behaviors including the selection of healthy foods and healthy eating patterns that motivate others (in the network) to change behaviors, and sometimes directly influence foods consumed or food related behaviors [12]. Behavior change may also be spread interpersonally within networks, wherein individuals observing behaviors of others in their network may have a higher probability of adopting similar behaviors [13].

South Asians are one of the fastest growing ethnic groups in the United States. Previous studies have shown higher risk of cardiovascular disease (CVD) and diabetes among South Asians [14, 15], and there is a paucity of evidence documenting effective interventions to improve diet quality and lower their risks. Current intervention programs focus on changing individual behaviors and based on western biomedical models and diets are not effective at reaching South Asians for several reasons. The vast majority of South Asians are immigrants who follow distinct dietary patterns with strong family and community structures that reinforce existing customs and norms related to foods [16]. Yet, little is known about the influence of social relationships on diet quality and dietary patterns in this high-risk group. Filling knowledge gaps about potential network drivers of diet quality in South Asians can help investigators develop more effective dietary interventions. Therefore, the overall goal of our study was to examine the correlations between social networks and diet quality examined using dietary patterns among South Asians in the U.S..

## Methods

### Study design, setting and participants

We used data from the Mediators of Atherosclerosis in South Asians Living in America (MASALA) study, which is a community based cohort of South Asians residing in the U.S. The detailed study information can be found elsewhere [17]. Briefly, 906 middle- to older- aged South Asians with no existing CVD were recruited at baseline using surname-based recruitment methods between 2010 and 2013 in the San Francisco Bay Area and the greater Chicago area. Baseline data were collected through interviews and clinical examinations. All surviving participants were re-contacted between 2014 and 2018 for an in-person ancillary study on social networks, and 771 participants completed the social networks visit. Interviewers also administered a Food Frequency Questionnaire (FFQ) at the social network visit. We excluded individuals who had incomplete information on dietary intakes (*n* = 1) and had implausible energy intake (< 800 or > 4200 kcal/day for men, < 500 or > 3500 kcal/day for women, *n* = 14), leaving a total of 756 participants who were included in these analyses.

### Measurement of personal social network characteristics

Data were collected from MASALA participants (herein called egos) using a social network module which included standard network elicitation as in previous work [18] and name generator instruments to collect egocentric network data. The surveys were administered in English, Hindi, or Urdu by trained interviewers. These network modules included a name generator that asked the names of people in the participants’ social network (herein called alters). Participants were asked to list up to 10 alters with whom they discuss “important matters” using a name generator which was previous used by the General Social Survey [19] and the National Social Life, Health, and Aging Project’s social networks module [20]. Network size was determined by the number of alters named by each participant in response to the name generator question. This name generator was designed to elicit network members who are close confidants (people with whom important information can be shared) and who may be socially influential. Name interpreter items followed and elicited information on the characteristics of alters, the relationship between egos and alters, and the egos’ perceptions of alters’ behaviors. For the first five alters, additional name interpreter questions obtained information about the alters’ socio-demographics (e.g. race/ethnicity and how many years living in the U.S.), type of relationship (e.g. spouse, child, friend), duration and strength of relationship, and frequency of communication. Name interpreter questions were limited to the first five alters to reduce ego burden which is a standard approach used in social network surveys [19, 20].

Egos were also asked about the dietary behaviors of their first five alters, including how often (‘never, a few times a year, monthly, weekly, daily’), over the 12 months, the alter consumed South Asian foods, fruits, vegetables, brown rice/quinoa, processed meat, sugar sweetened beverages, diet drinks, South Asian sweets, fried foods, fast foods, processed or packaged food. Egos also had the option of selecting “Don’t know”, and selected this response between 0.36% (12 respondents, for the question on consumption of South Asian foods) and 4.9% (161 respondents, for the question on consumption of brown rice/quinoa) of the time.

### Organizational affiliation

South Asians in the U.S. have developed organizational structures that may also exert a strong influence on health behaviors. We developed a roster of South Asian organizations (religious, social, cultural, community-based organizations) in Chicago and the San Francisco Bay area using key informant input and an iterative approach (described previously [21]). Egos were asked to look through the pre-defined list and select organization(s) that they visited within the prior 12 months and the frequency with which they visited the organization in the prior 12 months. Respondents could choose multiple organizations if applicable, and they were also given the option to add an organization if it was not on the roster. There was no limit on the number of organizations a person could affiliate with.

### Measurement of dietary intake and derivation of dietary patterns

The ego’s dietary intake at the social network visit was assessed with the Study of Health Assessment and Risk in Ethnic groups (SHARE) FFQ, which was developed for dietary assessment of South Asians in North America, has been previously validated among South Asians in Canada [22] and was used at baseline to assess dietary intakes of the cohort. A total of 161 food items was included in the SHARE FFQ with 61 food items specific for South Asian diet, and food items were additionally classified into 29 food groups, including added fat, alcohol, butter/pure ghee, coffee, eggs, fish, fried snacks, fruits, fruit juice, high-fat dairy products, high-sugar drinks, legumes/daal/tofu, low-fat dairy products, low-sugar drink, margarine, nuts and peanut butter, pasta, pizza, potatoes, poultry, red meat, refined grains, rice, snacks, Indian sweets and non-Indian desserts, tea, vegetable oil, vegetables, and whole grains. The SHARE FFQ assessed the dietary intakes including frequency and serving size of participants in the past year. Egos could choose from three options for serving size, which were small, medium and large, and small and large serving sizes were converted to medium serving size scale by multiplying by 0.5 and 1.5, respectively, in the analysis. Energy and nutrient intakes were derived using the ESHA Food Processor nutrient analysis software version 6·11 (1996).

As has been done previously in the cohort [23], foods on the FFQ were categorized into 29 food groups based on their similarity, nutrient composition, and culinary use in South Asian diets. Principal component analyses (PCA) with varimax rotation was then performed on the correlation matrix of the 29 food groups to derive dietary patterns. A 3-factor solution which explained 23% of the total variance of food intake was selected after evaluation of factor solutions with eigenvalues > 1 and examination of scree plots. Participants were assigned a factor score for each dietary pattern based on the linear combination of his or her FFQ data with the factor loadings in the 3 prevalent patterns. Food groups with factor loadings > 0.2 were considered a significant contributor to the dietary pattern, and the three dietary patterns were named based on food groups with highest factor loadings.

### Covariates

Covariates were selected based on previous literature and univariate analysis and included egos’ demographic characteristics such as age, sex, marital status, smoking status, annual income (≥$75,000 vs. <$75,000), education (≥ Bachelor’s degree vs. < Bachelor’s degree) all assessed by structured interview. Self-rated health was analyzed on a continuous scale ranging from 1 to 10, where 1 indicates poor health and 10 indicates excellent health. Participants were classified as having strong, moderate or weak traditional South Asian cultural beliefs according to a traditional cultural beliefs scale previously developed and validated in the cohort [24]. Daily energy intake was estimated from FFQ and analyzed on a continuous scale. BMI was calculated from measured weight in kilograms divided by square of height in meters. Information on social network size was derived using data from social network interview, and because detailed information was only obtained from first 5 alters, we adjusted the social network size with the maximum of 5. For individuals born outside the U.S., length of residency in the U.S. was asked and the percentage of time that living in the U.S. was calculated. Intentional exercise was assessed by the Typical Week’s Physical Activity Questionnaire, including activities such as walking for exercise, dance, conditional activities and sports, and total metabolic equivalent minutes per week were used for analysis [25]. While age, self-rated health, daily energy intakes and social network size were from the social network visit; sex, marital status, smoking status, BMI, income, education, study site, and traditional South Asian cultural beliefs were from the baseline visit.

### Statistical analysis

Socio-demographic characteristics were reported with means and standard deviation (SD) or percentage. The most basic measure of personal network structure was degree: the number of network members reported. The remaining network variables were calculated using information on the first five alters listed in response to the name generator question. The proportion of the social network members with each dietary behavior was calculated and used for analysis. We also calculated the average number of organizational affiliations for each participant.

We used residual diagnostics to assess the linearity of the relationship between dietary pattern scores and perceived dietary intake of network members. Partial correlations were used to assess the correlation between dietary pattern factor score and social network characteristics adjusting for ego age, gender, study site, education, income, marital status, traditional cultural beliefs, caloric intake, social network size, self-rated health, intentional exercise and percent life in the U.S.. When calculating correlations, network characteristics and dietary pattern factor scores were analyzed in their original (continuous) scale in order to better preserve relationships between dietary patterns and network characteristics and also to avoid the loss of statistical power that would be the result of collapsing continuous data into discrete categories.

All statistical tests were performed using two-sided tests with α = 0.05 and were conducted using SAS, version 9.4 (SAS Institute; Cary, NC).

## Results

The socio-demographic characteristics of the MASALA egos are shown in Table 1. The mean age of the study sample was 59(SD = 9) y with 44% women. Eight-9 % of participants had at least Bachelor’s degree, and only 3% were current smokers. The mean BMI was 26.5(SD = 4.1) kg/m^2^. The mean social network size was 5.5(SD = 2.6), and the mean age of alters was 50(SD = 9) y with 55% of alters being women. Participants’ networks had a high proportion of family (72%) and South Asians (89%).

**Table 1:** Socio-demographic characteristics among MASALA participants (egos) and their social network (alters)

	Mean ± SD or n (%)
N	756
Age, y	59.1 (9.2)
Women	336 (44.4)
NWU site	317 (41.9)
Education, Bachelor’s degree or above	675 (89.3)
Income (> = 75,000), *n* = 734	558 (76.0)
Has spouse or co-resident partner	675 (89.3)
Current smoker	26 (3.4)
Traditional cultural beliefs, *n* = 755	
Strong	140 (18.5)
Intermediate	423 (56.0)
Weak	192 (25.4)
Body mass index, kg/m^2^ *n* = 749	26.5 (4.1)
Self-rated health, ^a^ *n* = 754	7.8 (1.4)
Energy intake, kcal	1620 (487)
Percent of Life in the U.S.	50.0 (18.4)
Intentional exercise, MET-min/week, *n* = 755	1527 (1434)
Social network size (number in network analysis)	5.5 (2.6)
Social network attributes	
Network age, y	50.1 (9.2)
Proportion Women	0.55 (0.26)
Proportion Family members	0.72 (0.28)
Years living in the US	29.7 (10.0)
Proportion South Asian ethnicity	0.89 (0.23)

Abbreviations: *NWU* Northwestern University, *SD* Standard Deviation

^a^Self-rated health was analyzed on a continuous scale ranging from 1 to 10, where 1 indicates poor health and 10 indicates excellent health

Table 2 shows the factor loadings of each food group and variance explained for the three dietary patterns determined by principal component analysis. Based on the food group factor loadings, three dietary patterns were named: animal protein pattern characterized by higher intakes of alcohol, coffee, eggs, animal protein, pasta, pizza and refined grains; fried snacks, sweets and high-fat dairy pattern characterized by higher intakes of added fat, butter/*ghee* (clarified butter), fried and non-fried snacks, high fat dairy, legumes, potato, grains and sweets; and a fruits, vegetables, nuts and legumes pattern characterized by higher intakes of fruits, legumes, nuts, vegetable oil and vegetables.

**Table 2:** Ego food group factor loadings and variance explained for three dietary patterns assessed by principal component analysis

	Animal protein	Fried snacks, sweets and high-fat dairy	Fruits, vegetables, nuts and legumes
Added fat	–	0.24	0.21
Alcohol	0.29	–	–
Butter/ghee	–	0.27	–
Coffee	0.23	–	–
Eggs	0.28	–	–
Fish	0.34	–	–
Fried snacks	–	0.33	–
Fruit	–	–	0.49
Fruit juice	–	–	–
High fat dairy	–	0.20	–
Sugar-sweetened beverages	–	–	–
Legumes	−0.21	0.31	0.26
Low fat dairy	–	–	–
Low sugar drinks	–	–	–
Margarine	–	–	–
Nuts	–	–	0.31
Pasta	0.20	–	–
Pizza	0.23	–	–
Potatoes	–	0.32	–
Poultry	0.38	–	–
Red meat	0.42	–	–
Refined grains	0.21	0.22	–
Rice	–	0.40	–
Snacks	–	0.21	–
Sweets	–	0.31	–
Tea	–	–	–
Vegetable oil	–	–	0.35
Vegetables	–	–	0.53
Whole grain	–	–	–
Variance Explained (%)	9.3	7.4	6.5

*Note*: Absent scores represent factor loadings < 0.2

The correlations between social network characteristics and egos’ dietary patterns are shown in Table 3. Animal protein pattern scores were positively correlated with the number of years that alters lived in the U.S. (*r* = 0.111 *P* = 0.003) and negatively correlated with several network characteristics including the proportion of network who were family (*r* = − 0.109 *P* = 0.003), of South Asian ethnicity (*r* = − 0.258 *P* < 0.001) and the number of organizational affiliations (*r* = − 0.226 *P* < 0.001). Scores of the fried snacks, sweets and high-fat dairy pattern were positively correlated with the proportion of network that was South Asian (*r* = 0.093 *P* = 0.013). There were no statistically significant associations between the Fruits, vegetables, nuts and legumes pattern with general social network characteristics.

**Table 3:** Partial correlations between social networks and dietary patterns in the MASALA cohort

	n	Animal protein		Fried snacks, sweets and high-fat dairy		Fruits, vegetables, nuts and legumes	
		Partial Correlation Coefficient	*P*	Partial Correlation Coefficient	*P*	Partial Correlation Coefficient	*P*
*Network characteristics*							
Network Size	729	0.034	0.370	− 0.001	0.978	0.012	0.749
Mean age within network	729	0.007	0.850	− 0.016	0.674	0.062	0.098
Mean number of years living in the US	728	0.111	**0.003**	−0.055	0.143	0.023	0.544
Proportion of network is family member	729	−0.109	**0.003**	0.009	0.814	0.033	0.379
Proportion of network is South Asian	729	−0.258	**<.001**	0.093	**0.013**	0.042	0.261
*Perceived dietary intakes*							
Proportion of network:							
Is vegetarian	729	−0.505	**<.001**	0.169	**<.001**	0.054	0.147
Eats SA food daily	729	−0.225	**<.001**	0.141	**<.001**	0.022	0.551
Eats non-SA food daily	728	0.288	**<.001**	−0.124	**<.001**	−0.010	0.790
Eats raw vegetables daily	725	0.037	0.329	−0.134	**<.001**	0.176	**<.001**
Eats cooked vegetables daily	727	−0.120	**0.001**	−0.004	0.921	0.092	**0.014**
Eats fruit daily	728	−0.091	**0.015**	−0.027	0.468	0.118	**0.002**
Eats brown rice/quinoa daily/weekly	727	−0.035	0.352	−0.118	**0.002**	0.156	**<.001**
Eats non-vegetarian daily/weekly	729	0.510	**<.001**	−0.144	**<.001**	−0.067	0.073
Eats processed meat daily/weekly	727	0.330	**<.001**	−0.021	0.583	−0.051	0.174
Network drinks SSB daily	727	0.039	0.296	0.090	**0.016**	−0.079	**0.034**
Drinks diet drinks daily	723	0.103	**0.006**	−0.005	0.901	0.026	0.485
Eats SA sweets daily/weekly	729	−0.039	0.298	0.165	**<.001**	−0.125	**<.001**
Eats fried foods daily/weekly	728	0.086	**0.022**	0.128	**<.001**	−0.098	**0.009**
Eats fast food daily/weekly	729	0.012	0.744	0.149	**<.001**	−0.059	0.116
Eats out at restaurants (not fast food) daily/weekly	727	0.120	**0.001**	−0.015	0.691	−0.034	0.364
Adds *ghee* to food daily/weekly	727	−0.149	**<.001**	0.208	**<.001**	−0.015	0.682
Network eats processed/packaged foods daily	727	0.002	0.958	0.017	0.659	−0.010	0.781
Number of organizational affiliations	729	−0.226	**<.001**	0.083	**0.027**	0.018	0.625

Abbreviations: *SA* South Asian, *SSB* Sugar-sweetened beverages

*Note*: Model adjusting for age, sex, marital status, income, education, study site, self-rated health, traditional cultural beliefs, energy intake, intentional exercise, percent life in the U.S. and social network size

Perceived dietary intakes of alters were also significantly correlated with dietary patterns of the egos. Animal protein pattern tend to have stronger correlations with perceived dietary intakes of network members compared to the other two patterns. Specifically, there were significant correlations between the animal protein pattern and the proportion of the network that consumed non-vegetarian foods (*r* = 0.510), non-South Asian foods (*r* = 0.288) and processed meat (*r* = 0.330) daily or weekly. For the fried snacks, sweets and high-fat dairy pattern, the adjusted correlations ranged from − 0.118 for the proportion of the network consuming brown rice/quinoa daily or weekly to 0.208 for the proportion of network that added *ghee* to food daily or weekly. For fruits, vegetables, nuts and legumes pattern, the adjusted correlations ranged from − 0.079 for the proportion of the network consuming sugar sweetened beverages daily to 0.176 for perceived network intakes of raw vegetables daily (Table 3).

## Discussion

Social network characteristics and perceived dietary intakes and behaviors of social network members are correlated with dietary patterns in a community-based cohort of middle aged and older South Asians in the U.S.. Higher fruits, vegetables, nuts and legumes dietary pattern scores of egos were associated with a higher proportion of networks consuming vegetables and fruit daily and brown rice or quinoa. Higher scores on the animal protein pattern were associated with a higher proportion of network members following a non-vegetarian style diet and consuming processed meat, diet drinks and fried foods. Finally, higher scores on the fried snacks, sweets and high-fat dairy pattern were associated with a higher proportion of networks consuming more sugar-sweetened beverages, *ghee*, South Asian sweets, fried and fast foods and less brown rice or quinoa.

Three dietary patterns were identified in the MASALA cohort. The animal protein pattern was characterized by higher intakes of meat and westernized foods such as pasta and fast foods, which indicates dietary transition and adaption to the westernized foods after immigration. Higher scores on the animal protein patterns were associated with a higher proportion of networks with longer residency in the U.S., who were not family members or of South Asian ethnicity. Network members who have lived in the U.S. longer and who were not South Asian may have more western dietary habits which influence the ego’s dietary patterns. Egos who were affiliated with fewer South Asian organizations also had higher scores on the animal protein pattern, which may be a marker for being more acculturated and less involved in South Asian cultural or religious events. This might result in having a more westernized lifestyle and adopt western eating pattern. The fried snacks, sweets and high-fat dairy pattern was characterized by higher intakes of both fat and carbohydrates, and it captured a more traditional South Asian dietary pattern with less healthy foods; higher scores for this pattern was associated with networks with a higher proportion of South Asians and those who have lived in the U.S. for fewer years, consumed South Asian foods daily, and were vegetarian.

The significant correlations between perceived dietary intakes of networks and dietary patterns of MASALA study participants indicates interpersonal ties may influence dietary intakes, and that information on eating habits or foods may be transmitted between networks and egos. Individuals may be influenced and therefore adopt dietary behaviors of networks around them by observing the dietary behaviors and attitudes of their network members. It may also change participant’s norms by increasing the tolerance and changing the perception of eating unhealthy foods if network members follow unhealthy dietary patterns [26]. Therefore, individuals may accept consumption of unhealthy foods if they know their networks consume unhealthy foods as well. This study also demonstrated that egos were likely to have dietary patterns similar to their networks members.

Our findings on significant correlations between the fruits, vegetables, nuts and legumes pattern and network intakes of fruits and vegetables were consistent with previously published literature. In a study using data from the Harvard Cancer Prevention Program Project, individuals whose family and friends met the standard of consuming at least 5 servings of fruits and vegetables per day were more likely themselves to consume at least five servings of fruits and vegetables per day [27]. It is important to note that it is not just healthy habits or dietary patterns that may be transmitted between egos and their social networks; in a cross-sectional analysis of dietary behaviors among middle-aged mostly African American public housing women residents showed higher daily added sugar consumption was significantly associated with higher proportion of their networks who had higher daily sugar-sweetened beverage and sweets consumption [12]. Our study supports this finding since there were correlations between perceived consumption of sugar-sweetened beverages, fried foods, fast foods and the less healthy fried snacks, sweets, and high fat pattern in this South Asian cohort, as well as the animal protein pattern and network consumption of processed meat.

The role and influence of social networks on dietary behaviors is an important factor that should not be neglected in lifestyle intervention programs. In a community participatory research project including men and women in the Brazilian Amazon for reducing methylmercury from fish consumption, dietary behavior change for fish intake was associated with increased numbers of discussion partners whom participants could talk with about adoption new fish intake behavior [28]. The *Entre Familia*: *Reflejos de Salud* Study also showed that sharing program materials on how to improve dietary behaviors with others was associated with an increase in fiber intake among U.S. Latino families [29]. Dietary behavior change caused by interpersonal interaction within social networks may be explained by the Diffusion of Innovations Theory and Social Comparison Theory [30]. The Diffusion of Innovation Theory focuses on the spread of new ideas within networks, and supports the idea that individuals are more likely to adopt new healthy dietary behaviors if their network members discuss these with them, and individuals are more likely to change their behaviors if they share similar culture, traditions, health practice and environment with their networks [31]. The Social Comparison Theory explains self-evaluation by comparing oneself with others, especially among individuals within a social network. Because network members may have similar values and knowledge, individual behaviors or attitudes towards healthy eating may change or be maintained via communication with or observation of network members behaviors since individuals tend to conform to others in the social group in order to keep appropriate behaviors within the social group [30].

The supportive role of social network members on encouraging healthy eating and healthy foods selection is also important for dietary behavior change. Families and close friends have an influential role on dietary intake because they often eat together and comments on food selected and consumed can be frequent, and individuals can be more motivated on behavior change by the support of families and friends. We recently reported that positive role modeling and support from adult children were important facilitators of healthy dietary changes in older South Asians, suggesting that adult children may be influential network members in South Asian families [32]. Results from the Mexican American mother-daughter dyads of *Unidas por la Vida* intervention, demonstrated that participants in the intervention group tend to have low glycemic load diet and consume less saturated fat which may be explained by the greater health-related social support and social control persuasion from their network members [33]. Also, intervention program participants can influence others to achieve dietary behavior change. In the Healthy-Living Partnerships to Prevent Diabetes study, participants in the intervention group indicated better perceived change on dietary and physical activity behaviors among their network members who provided social support [34].

The strengths of this study include its well-characterized study population. To our knowledge, this is the first study of social networks and their association with dietary behavior in a large community-based South Asian cohort in the U.S., a population that is at high-risk of lifestyle related chronic conditions. Dietary data were collected using a validated and culturally appropriate FFQ. Limitations include the cross-sectional nature of these analyses which prohibits the ability to conclude causal relations. Second, social network characteristics were based on egocentric data, so the perceived information of network members on relationships and dietary behaviors may not be accurate; however, others have shown that perceptions are as or maybe even more important than actual behavior of network members in influencing a respondent’s behavior [35]. Third, the correlations between network characteristics and dietary patterns ranged from 0.001 to 0.5, and the magnitude of some of these were small; however, being one of the first investigations that has examined the influence of social network characteristics in a South Asian immigrant population justifies the focus of this study. Future research needs to focus on identifying key network characteristics and individuals who may exert the most influence on dietary behaviors of egos. Lastly, the cohort included middle-older aged South Asians, and most of them had higher level of education, therefore our findings may not generalizable to younger or less educated South Asian immigrants.

## Conclusions

This study showed that social network characteristics and perceived dietary behaviors of network members were correlated with dietary patterns of South Asian immigrants in the U.S. Dietary behaviors may spread within social network group and assist in behavior change. Dietary intervention studies among South Asians should consider social network characteristics as candidate components for dietary intervention.

## Data Availability

The datasets generated and/or analysed during the current study are not publicly available but are available from the corresponding author on reasonable request.

## Abbreviations

CVD: Cardiovascular Disease
MASALA: Mediators of Atherosclerosis in South Asians Living in America
FFQ: Food Frequency Questionnaire
SHARE: Study of Health Assessment and Risk in Ethnic groups
SD: Standard Deviation
